# RHOQ is induced by DLL4 and regulates angiogenesis by determining the intracellular route of the Notch intracellular domain

**DOI:** 10.1007/s10456-020-09726-w

**Published:** 2020-06-06

**Authors:** Esther Bridges, Helen Sheldon, Esther Kleibeuker, Evelyn Ramberger, Christos Zois, Alun Barnard, Ulrike Harjes, Ji-Liang Li, Massimo Masiero, Robert MacLaren, Adrian Harris

**Affiliations:** 1 Cancer Research UK Department of Medical Oncology, Weatherall Institute of Molecular Medicine, University of Oxford, Oxford, OX3 9DS UK; 2 Radcliffe Department of Medicine, NDCLS, Oxford, OX3 9DU UK; 3 Oxford Eye Hospital, University of Oxford, Oxford, OX3 9DS UK

**Keywords:** Angiogenesis, RHOQ, DLL4 Notch1 signalling, NICD degradation, Autophagy pathway

## Abstract

Angiogenesis, the formation of new blood vessels by endothelial cells, is a finely tuned process relying on the balance between promoting and repressing signalling pathways. Among these, Notch signalling is critical in ensuring appropriate response of endothelial cells to pro-angiogenic stimuli. However, the downstream targets and pathways effected by Delta-like 4 (DLL4)/Notch signalling and their subsequent contribution to angiogenesis are not fully understood. We found that the Rho GTPase, RHOQ, is induced by DLL4 signalling and that silencing RHOQ results in abnormal sprouting and blood vessel formation both in vitro and in vivo. Loss of RHOQ greatly decreased the level of Notch signalling, conversely overexpression of RHOQ promoted Notch signalling. We describe a new feed-forward mechanism regulating DLL4/Notch signalling, whereby RHOQ is induced by DLL4/Notch and is essential for the NICD nuclear translocation. In the absence of RHOQ, Notch1 becomes targeted for degradation in the autophagy pathway and NICD is sequestered from the nucleus and targeted for degradation in lysosomes.

**Electronic supplementary material:**

The online version of this article (10.1007/s10456-020-09726-w) contains supplementary material, which is available to authorized users.

## Introduction

Angiogenesis is a key component of the growth and metastasis of tumours [1]. Therapeutic approaches to target angiogenesis have presented definite but limited clinical benefits [2]. The regulation of angiogenesis is a balance between key promoting and inhibiting angiogenic signals. Following a pro-angiogenic stimulus, some of the endothelial cells (EC) lining the blood vessels differentiate into a tip cell phenotype. Tip cells migrate towards the source of the stimulus and are followed by EC that acquire a ‘stalk cell’ phenotype. Stalk cells proliferate to extend the growing sprout guided by the leading tip cell. One of the best defined pro-angiogenic factors is vascular endothelial growth factor (VEGFA), which promotes tip cell formation, migration, proliferation and suppresses apoptosis upon stimulation of VEGF receptor 2 (VEGFR2) [3].

The Notch signalling pathway plays an important role in cell fate and differentiation, and can modify cellular processes such as proliferation [3]. In EC biology Notch signalling is critical in ensuring the appropriate response of EC to various pro-angiogenic stimuli. EC Delta-like 4 (DLL4) expression is induced by VEGFA and activation of DLL4/Notch signalling also leads to an upregulation of DLL4 in a positive feed-forward mechanism. DLL4/Notch signalling regulates angiogenesis by influencing the differentiation of EC and promoting a stalk cell phenotype, partially though decreasing VEGFR2 surface expression and desensitising the cell to VEGF stimulus [4]. In tumour angiogenesis, interfering with DLL4/Notch signalling leads to reduced neoplastic growth, partially due to excessive EC tip cell formation and sprouting, ultimately resulting in poorly perfused and non-functional vessels not capable of sustaining efficient tumour growth [5].

The endothelial Notch ligand, DLL4 on the cell surface, binds to the Notch receptor on neighbouring cells (reviewed in [4]). This results in two proteolytic cleavages of the Notch receptor, internalisation of the receptor and subsequent release the Notch Intracellular domain (NICD) into the cytoplasm of the signal receiving cell. The NICD translocates into the nucleus, where it associates with the CSL complex to either initiate or repress transcription of Notch target genes (reviewed in [6]). Notch signalling can be regulated by endocytosis by sequestering the Notch receptor from the cell surface or by internalising the receptor/NICD into intracellular compartments (endosomes) leading to the degradation of receptor/NICD in lysosomes [7]. However, it is increasingly clear that there is another aspect of Notch signalling within the endosomes. This is referred to as the non-canonical pathway, by which other pathways can induce Notch signalling in a ligand independent mechanism within the cell (reviewed in [8]). Here, Notch receptors are internalised into early endosomes, which fuse into late endosomes and then into the multivesicular body. Proteins such as Deltex stabilise the Notch receptor within the compartments [9]. This protects the Notch receptor (and NICD from degradation) and enables cleavage of the Notch receptor and Notch signalling to occur. The process which regulate this bifurcation for NICD nuclear signalling and the balance between degradation is not fully understood, particularly within EC.

To further elucidate the molecular cascade induced by DLL4 on EC, we examined data from the FANTOM4 project (https://fantom.gsc.riken.jp/) where human umbilical vein endothelial cells (HUVECs) had been stimulated with immobilised human recombinant DLL4 (rhDLL4) for 16 h and deep sequence Cap Analysis Gene Expression (CAGE) analysis performed to identify novel DLL4/Notch downstream targets [10]. Among the most highly induced genes we identified RHOQ, also known as TC10.

RHOQ is a member of the small Rho GTPase family, which cycles between an inactive GDP and active GTP bound state [11]. Post-translational modifications of RHOQ targets the protein to lipid rafts/caveolae microdomains [12], or to actin filaments, the plasma membrane and vesicles [12–17]. For instance, active RHOQ interacts with Exo70, a component of a protein complex that transports various vesicles with specific cargoes, e.g., Glut1, Glut4 [12–15] or Rab11 to the cell surface [18]. RHOQ is also involved in membrane expansion in axonal regeneration processes [19] and in the formation of filopodia during neurite outgrowth [20–23]. Recently, RHOQ was shown to mediate upregulation of Glut1 following stimulation of VEGFR2 in mouse embryonic stem cells [24]. RHOQ is involved in the trafficking of CAL (cystic fibrosis transmembrane conductance regulator associated ligand) to the plasma membrane, thus protecting CAL from being degraded in lysosomes [25]. In addition, active RHOQ influences transcription mediated by nuclear factor kappaB, serum response factor and cyclin D1 by mechanisms not fully understood [11, 21].

However, the role of RHOQ as a downstream target of Notch/DLL4 signalling in endothelial cells during the angiogenic process is currently unknown. By utilising knockout and over expression studies in different models of angiogenesis both in vitro and in vivo*,* we demonstrate RHOQ is a critical ‘feed forward’ regulator of DLL4/Notch signalling and in determination of the fate of the NICD.

## Results

### RHOQ is induced by DLL4/Notch signalling in vitro

**Fig. 1:**
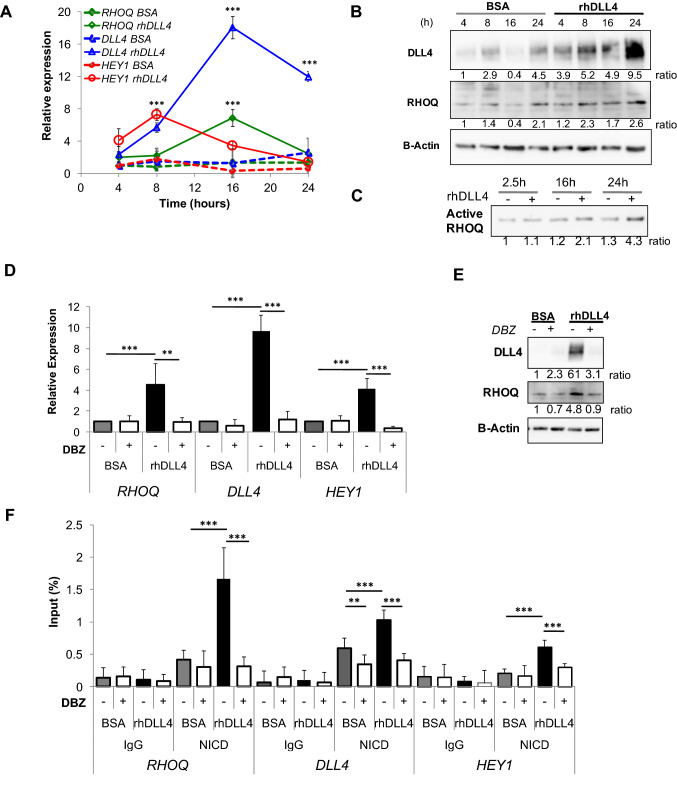
RHOQ is induced by DLL4/Notch signalling in vitro. HUVECs were grown on BSA or rhDLL4-coated plates, harvested at indicated time points and changes in RHOQ and DLL4/Notch targets were assessed by **a** QPCR or **b** by western blotting. **c** Active RHOQ status following GTPase pull down assay and magnitude of active RHOQ was detected by western blotting. Changes in DLL4/Notch target expression following 16 h incubation with DBZ (20 nM) compared to DMSO equivalent controls was assessed by either. **d** QPCR, normalised to BSA DMSO control, or **e** by western blotting, or **f** cells were fixed and analysed for NICD bound to Notch promoter binding site for RHOQ, compared to that of DLL4 and HEY1 by chromatin immunoprecipitation. β-Actin as a loading control and densitometry was performed on western blots. Densitometry ratios were expressed relative to the first BSA control sample (Error bars = S.D. Key: *p < 0.05, **p < 0.01, ***p < 0.0001 one-way ANOVA or unpaired Student’s *t*-test comparing two data groups; data representative of *n* = 3 independent experiments)

DLL4/Notch signalling was activated in HUVECs stimulated with tethered rhDLL4 [26]. *RHOQ* mRNA (Fig. 1a) and RHOQ protein (Fig. 1b) were induced in rhDLL4-stimulated HUVECs and the kinetics of induction mirrored that of *DLL4* mRNA and protein (although with smaller magnitude). Both transcripts peaked at 16 h and were preceded by rhDLL4 induced *HEY1* upregulation that peaked at 8 h (*p* < 0.001; Fig. 1a).

To assess the effect of DLL4 signalling on RHOQ activation, HUVEC were stimulated with rhDLL4 over 24 h and the active/GTP bound proportion of RHOQ was precipitated from the cell lysates using the PAK protein binding domain (PBD) fused to agarose beads. RHOQ was found to be constitutively active [11] in rhDLL4-stimulated HUVECs compared to basal HUVECs (Fig. 1c).

Blockade of Notch signalling with the γ-secretase inhibitor DBZ (20 nM) inhibited rhDLL4 induction of *RHOQ*, *HEY1* and *DLL4* (Fig. 1d) and RHOQ and DLL4 (Fig. 1e). To verify that RHOQ is a direct Notch target, we analysed a predicted RBPKκ-NICD binding site (GTGGGAA [27]) at − 218 bp in the *RHOQ* gene promoter and compared to promoter regions of *DLL4* and *HEY1* as positive controls. The ChIP of bound NICD, compared to IgG control antibody, showed significantly increased binding to *RHOQ*, *DLL4* and *HEY1* promoters in 16 h rhDLL4-stimulated cells (Fig. 1f); this was significantly inhibited by DBZ.

### RHOQ regulates endothelial sprouting and tube formation

**Fig. 2:**
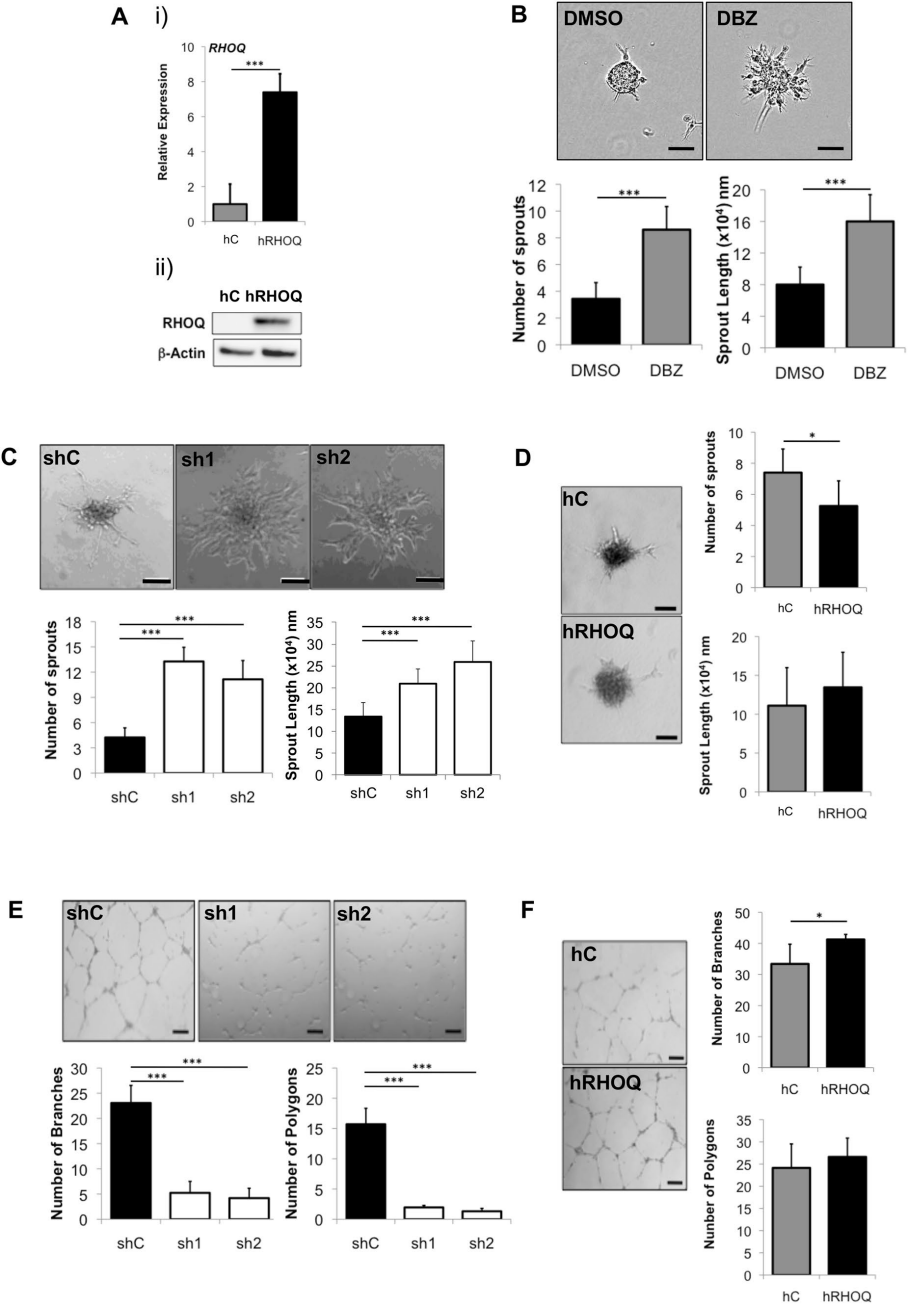
RHOQ is induced by DLL4/Notch signalling and regulates angiogenesis in vitro. **a** HUVECs were infected with control virus (hC) or human RHOQ (hRHOQ) expressing virus; RHOQ expression confirmed by (i) QPCR and expressed relative to the control cells or (ii) western blotting, using B-Actin as a loading control. **b** Phenotypic changes in the hanging drop sprouting assay 24 h following incubation with DBZ (20 nM) or DMSO control was assessed. Representative images and quantification of phenotypic changes in the hanging drop sprouting assay for **c** stable shRHOQ transfected or **d** human RHOQ (hRHOQ) overexpressing HUVECs compared to equivalent controls. Representative images and quantification of phenotypic changes in the tube formation assay for **e** stable shRHOQ transfected or **f** human RHOQ (hRHOQ) overexpressing HUVECs compared to equivalent controls (Error bars = S.D. Scale bar = 20 nm. Key: *p < 0.05, **p < 0.01, ***p < 0.0001 one-way ANOVA; data representative of n = 3 independent experiments)

To assess the role of RHOQ in angiogenesis, HUVECs were either transiently transfected with three small interfering (si) RNA specific duplexes targeting RHOQ (referred to as S1, S2, S3; Supplementary Fig. 1a) or infected with short hairpin RNA coding lentivirus to stably knockdown RHOQ (referred to as sh1 and sh2; Supplementary Fig. 1b). All three siRHOQ duplexes and stable knockdown resulted in a significant decrease in *RHOQ* mRNA (*p* < 0.001; Supplementary Fig. 1ai, bi) and RHOQ protein (Supplementary Fig. 1aii, bii) in both control and rhDLL4-stimulated HUVECs levels. Stable RHOQ overexpressing cells were also generated and increased *hRHOQ* mRNA (*p* < 0.001, Fig. 2ai) and hRHOQ protein (Fig. 2aii) was confirmed.

Disruption of Notch signalling by DBZ resulted in a significantly increased number and length of sprouts by EC spheroids in the hanging drop assay in vitro compared to control EC sprouting (Fig. 2b). A similar hyper-sprouting phenotype, with sprout number and length significantly increased, was also observed for both transient (*p* < 0.001, Supplementary Fig. 1c) and stable knockdown (*p* < 0.001, Fig. 2c) of RHOQ. Consistently, a significant decrease in the number of sprouts was observed in hRHOQ overexpressing HUVECs compared to controls (*p* < 0.05, Fig. 2d), although there was no significant difference in the length of sprouts (*p* > 0.05).

In the Matrigel assay [28] control HUVECs formed a coordinated arrangement of interconnecting enclosed areas (polygons) and branch points (more than 3 cells interconnecting) over an 8 h time period. Loss of RHOQ led to a loss of network formation, with few polygons and branch points formed at 8 h, in both transient (*p* < 0.001, Supplementary Fig. 1d) and stable (*p* < 0.001, Fig. 2e) RHOQ knockdown. Overexpressing hRHOQ increased the number of branch points formed, although there was no significant difference in overall polygon formation (*p* > 0.5; Fig. 2f).

### Reducing RHOQ expression compromised blood vessel formation with in the CAM assay

**Fig. 3:**
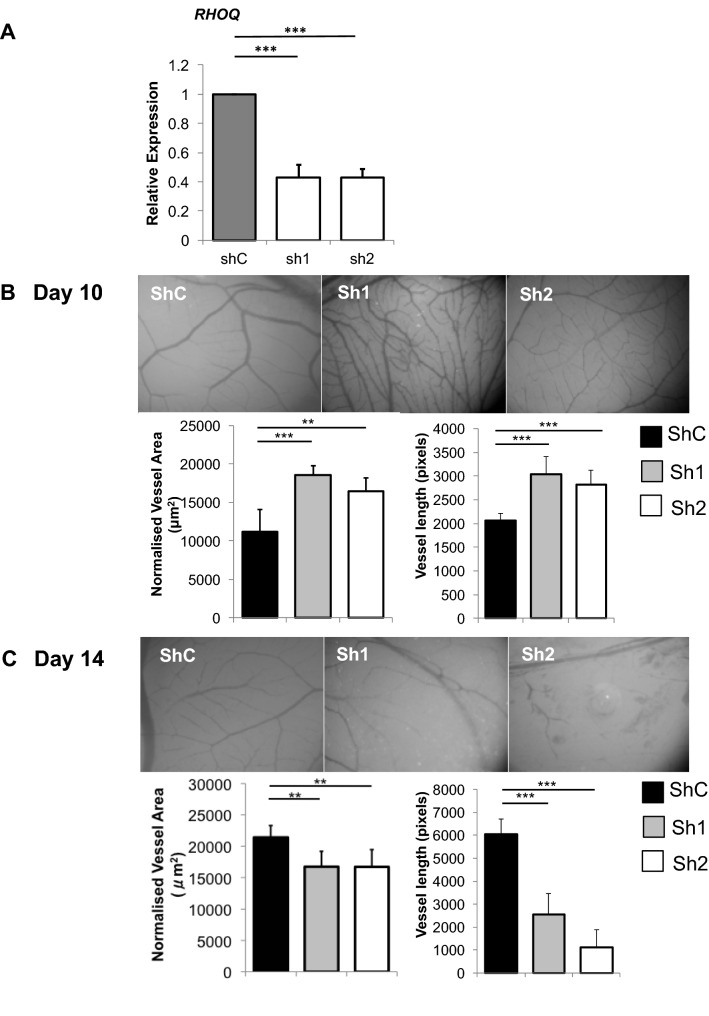
RHOQ is induced by DLL4/Notch signalling and regulates angiogenesis in vivo. **a** Chicken fibroblasts were infected with shControl (shC) virus or RHOQ shRNA duplexes (sh1, sh2) containing virus for stable loss of RHOQ and harvested after 24 h to confirm changes in RHOQ expression by QPCR. Representative images and analysis of vasculature changes on **b** EDD10 and **c** EDD13.9 following infection with shC virus or viruses containing RHOQ shRNA duplexes (Sh1, Sh2) on EDD3 in the chicken CAM assay (Error bars = S.D. Scale bar = 20 nm. Key: *p < 0.05, **p < 0.01, ***p < 0.0001 one-way ANOVA; data representative of *n* = 3 independent experiments)

The chicken CAM assay was utilised to study the role of RHOQ on developmental angiogenesis. We demonstrated that the shRHOQ lentivirus caused a 70–80% reduction in chicken *RHOQ* (Fig. 3a) and that human RHOQ could be expressed by in chicken fibroblasts (Supplementary Fig. 2a) after lentivirus exposure. CAMs were then infected with lentivirus on egg development day (EDD)3. Interestingly, both loss of chicken RHOQ (Fig. 3b) or overexpression of human RHOQ (Supplementary Fig. 2b) initially caused the formation of a denser vasculature network, as indicated in pictures and analysis of vasculature development at EDD10. By EDD13.9, modulation of RHOQ in both instances ultimately resulted in the regression and formation of abnormal vessel network (Fig. 3c, Supplementary Fig. 2c). Analysis of the effect of modulating RHOQ on EDD13.9 confirmed a significant decrease in vessel area coverage and length (Fig. 3c, Supplementary Fig. 2c).

### Reducing RHOQ expression compromised blood vessel formation with in the mouse retina assay

Vasculature in the postnatal retinal model develops within a short time frame in a highly stereotypic manner [29]. The expression profile and function of DLL4 and Notch1 has been extensively studied using this model and fluctuates during the key stages of developing vasculature [30–32]. DLL4 and Notch1 expression is observed in the immature capillaries and at the vascular front (containing EC tip cells) of the primitive vascular plexus that emerges from the central vessels at postnatal day P3. The superficial vasculature formed begins to mature by P6. From this time point DLL4 expression is more confined to arteries and Notch signalling becomes more sporadic and remains more central [30–32].

**Fig. 4:**
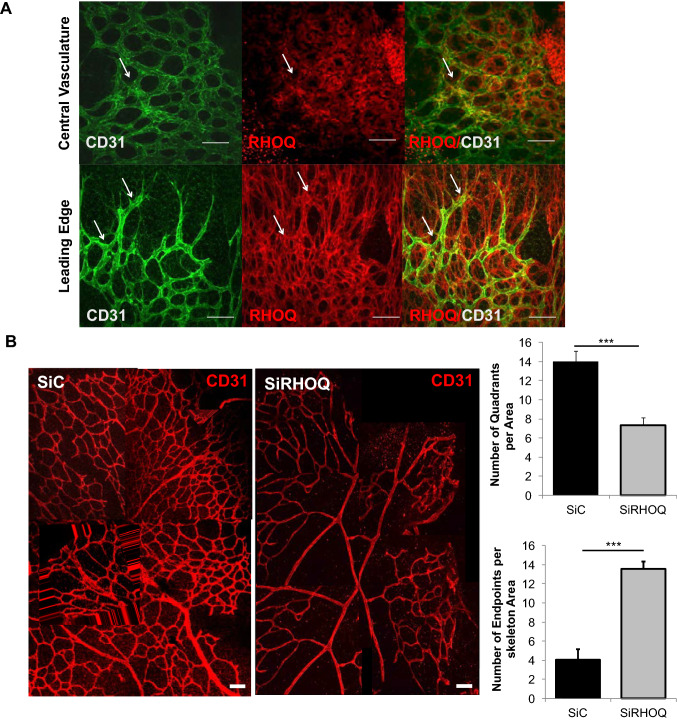
Loss of RHOQ disrupted retinal vasculature formation. **a** RHOQ (red) is expressed in endothelial cells (CD31, green), and other cell types, in the central vasculature and at the leading edge in retinas obtained from day 6 old pups. **b** Retinas were transfected following intraocular injection of 500 nM in 1 ul of SiControl (SiC) or SiRHOQ mouse duplexes using the in vivo GFP-labelled JETPEI delivery system on day 3 following birth of pups and eyes harvested on day 6 following birth and effects on vasculature assessed by immuno-fluorescence staining by analysis of quadrants formed at both central and leading edge vasculature and open endpoints of cells that failed to form a connection with quadrant in the central vasculature. (White arrow, example of co-localisation; Scale bar = 20 nm; data representative of *n* = 3 independent experiments)

Reflective of the Notch signalling expression profile at P6 [30–32], RHOQ was found strongly expressed in maturing endothelial cells located more centrally and in the newly formed vessels in the leading edge, as well as other cell types (Fig. 4a). The effect on vasculature following loss of RHOQ was evaluated by injecting GFP-labelled JETPEI lipofectamine-based vesicles containing 500 nM of siRNA targeting RHOQ into the retina on P3 and retinas examined three days later (thus loss of RHOQ occurring across the time period when Notch signalling is at its peak); RHOQ expression within the retina was reduced in different cell types including EC (Fig. 4b). Reduced RHOQ resulted in disorganised, pruned and fragmented vasculature networks compared to control vasculature (Fig. 4b). This was confirmed by analysis of number of quadrants, with loss of RHOQ resulted in more incomplete quadrants that had more open endpoints (where vessel sprouts failed to join to form a quadrants) compared to the control siC duplexes (Fig. 4b).

### Inhibiting RHOQ expression blocks transcription of DLL4/Notch target genes

Silencing of RHOQ results in a hyper-sprouting phenotype which suggests the DLL4/Notch signalling pathway is compromised. We therefore examined the status of the DLL4/Notch signalling in siRHOQ transfected EC.

**Fig. 5:**
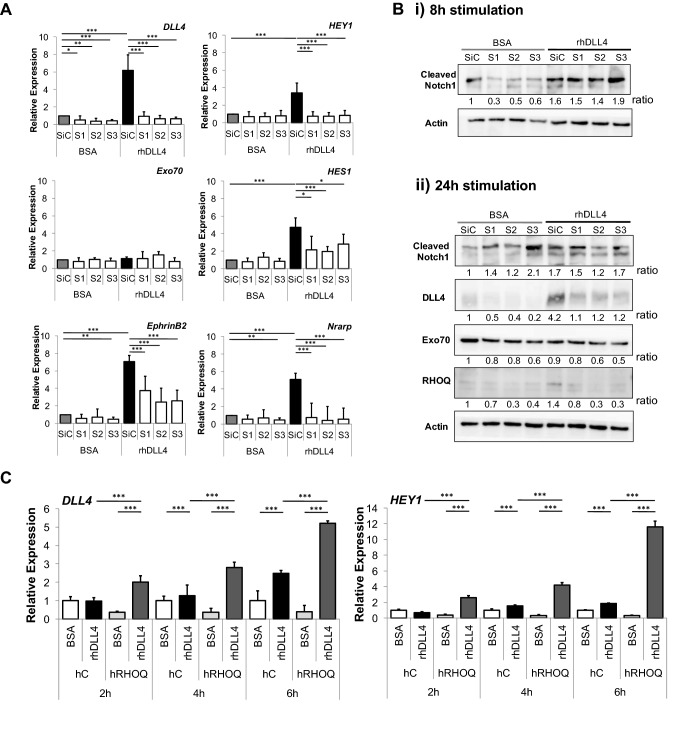
Loss of RHOQ expression abolished expression of downstream DLL4/Notch signalling. HUVECs were either transfected with siRHOQ or infected with human RHOQ (hRHOQ); cells were then grown on BSA or rhDLL4-coated plates and harvested at indicated time points. Changes in DLL4/Notch downstream target expression in stimulated siRHOQ transfected HUVECs were assessed by **a** QPCR after 16 h stimulation, expressed relative to BSA control, and **b** western blotting, after either (i) 8 h or (ii) 24 h stimulation, using B-Actin as a loading control. **c** Changes in DLL4/Notch downstream target expression in stimulated HUVECs overexpressing hRHOQ was assessed over time by QPCR. Densitometry ratios were expressed relative to the first BSA control sample (Error bars = S.D. Key: *p < 0.05, **p < 0.01, ***p < 0.0001 one-way ANOVA; data representative of *n* = 3 independent experiments)

Surprisingly, siRHOQ inhibited the induction of DLL4/Notch downstream targets [26, 28, 33–37] *DLL4*, *HEY1*, *HES1*, *EphrinB2* and *NRARP* in rhDLL4-stimulated HUVECs (*p* < 0.001, Fig. 5a). Cleaved Notch1 was observed at 8 h (Fig. 5bi) and at 24 h (Fig. 5bii). However, basal and induced DLL4 protein expression was reduced in rhDLL4-stimulated siRHOQ negative cells (Fig. 5bii). Overexpression of hRHOQ initially promoted DLL4/Notch signalling, with increased *DLL4* and *HEY1* expression observed at earlier time points in rhDLL4-stimulated hRHOQ compared to controls (Fig. 5c).

### Inhibiting RHOQ expression reduces surface expression of DLL4 and Notch1, leading to Notch1 degradation

**Fig. 6:**
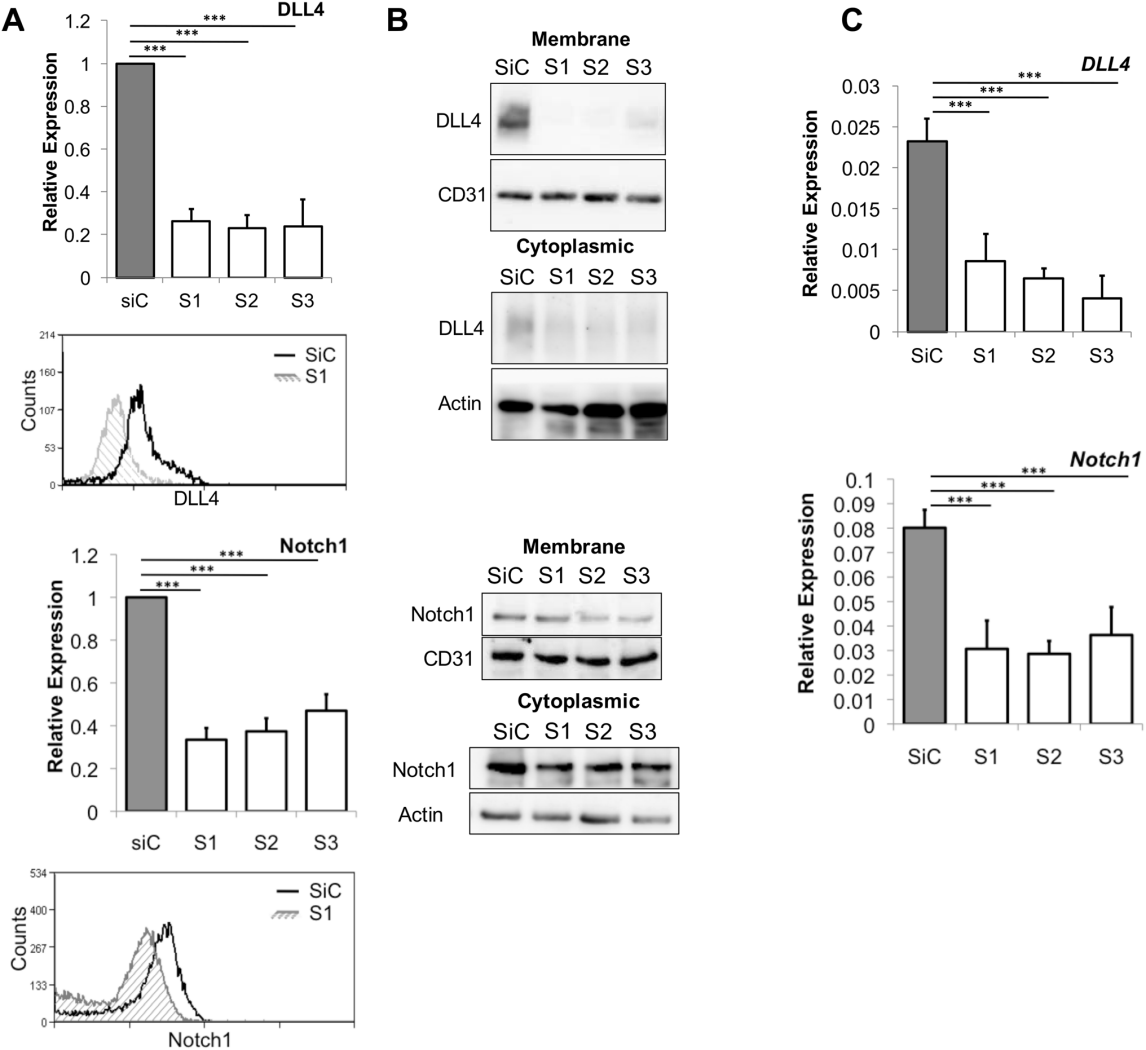
Loss of RHOQ reduces surface expression of Notch components. HUVECs were transfected with siRHOQ and the effects on DLL4 or Notch1 expression were assessed 24 h later by **a** staining cells for surface protein surface expression, analysed by FACs and expressed relative to control, with example representative image provided, **b** membrane fraction by western blotting, using CD31 as a loading control or **c** by QPCR, expressed relative to GAPDH (Error bars = S.D. Key: *p < 0.05, **p < 0.01, ***p < 0.0001 one-way ANOVA or unpaired Student’s *t*-test between data group and control; data representative of *n* = 3 independent experiments)

To determine at what point the Notch signalling pathway was compromised, we first investigated to see if DLL4 or Notch1 was still present on the cell surface following loss of RHOQ. A significant decrease in basal DLL4 surface expression was also observed in siRHOQ negative cells, assessed by both FACs (Fig. 6a) and membrane, cytoplasmic fraction analysis (Fig. 6b). Similarly, a significant decrease in basal *DLL4 RNA* within siRHOQ transfected cells was observed (Fig. 6c). The basal DLL4 expression levels remained unaltered on the cell surface of hRHOQ overexpressing cells following FACs analysis (Supplementary Fig. 3a). There were also no significant changes in DLL4 expression, membrane and cytoplasmic fraction analysis (Supplementary Fig. 3b) or basal *DLL4* mRNA (Supplementary Fig. 3c) in hRHOQ overexpressing cells.

Similarly, we found that basal Notch1 surface expression decreased as assessed by FACs (Fig. 6a) and by membrane fraction analysis (Fig. 6b), and *Notch1* mRNA was also significantly decreased (Fig. 6c). The basal expression levels of Notch1 remained unaltered on the cell surface of hRHOQ overexpressing cells by FACs analysis (Supplementary Fig. 3a) and there were no significant changes in membrane or cytoplasmic fraction analysis (Supplementary Fig. 3b) or *Notch1* mRNA levels (Supplementary Fig. 3c).

### Interaction of RHOQ and Exo70 with Notch1 and cleaved Notch1

RHOQ interacts with Exo70 as part of a protein complex that transports various vesicles to sites of secretion [12–15], as well as interacting with proteins bound for the nucleus [21, 24]. We investigated RHOQ and Exo70 interaction with Notch components. EC stimulated with rhDLL4 for 8 h were cells examined for changes in cleaved Notch1 (NICD) within the cytoplasm as well as the nuclear levels, before the NICD becomes rapidly degraded. The changes in Notch1 receptor localisation was compared to RHOQ and Exo70 at the previous experimental times of 16 h stimulation.

**Fig. 7:**
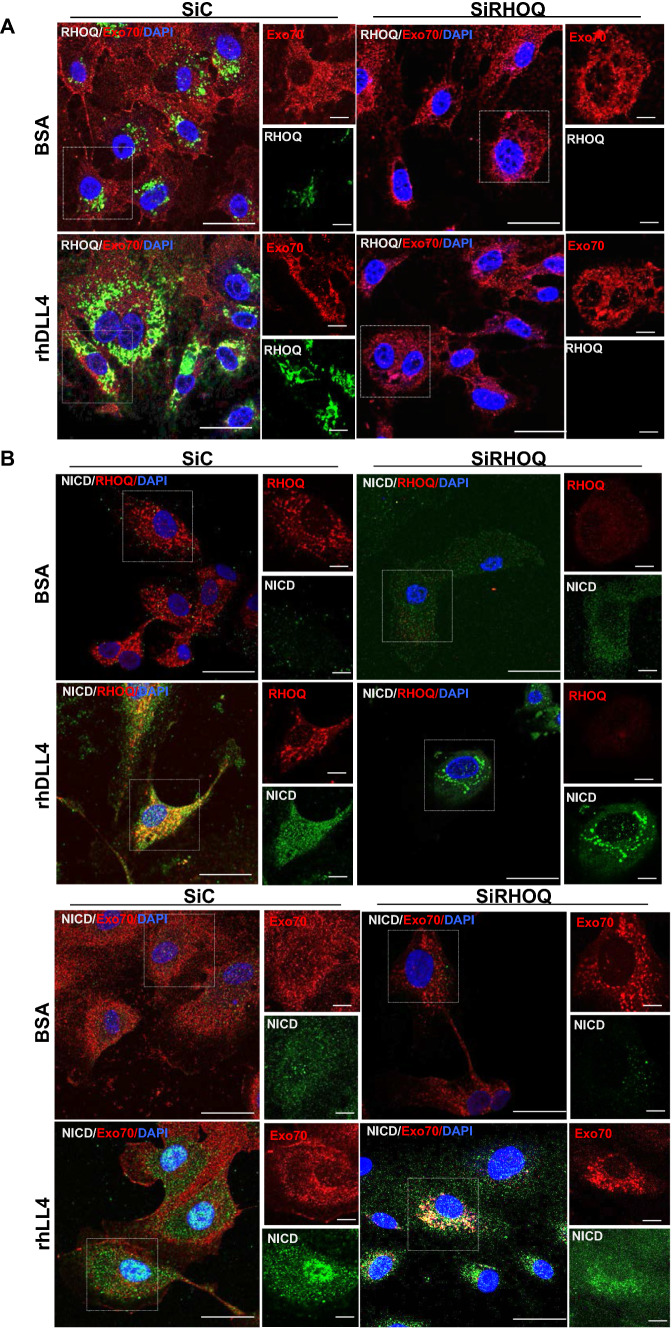
Localisation changes of NICD in RHOQ negative cells. **a** Transfected HUVECs with RHOQ siRNA duplexes were cultured on BSA or rhDLL4 (1 μg/ml)-coated plates for 8 h for NICD analysis or 16 h for other markers before immuno-fluorescence staining for **a** RHOQ (green) and Exo70 (red), **b** NICD (green) with RHOQ (red) or Exo70 (red) and visualised by confocal microscopy. Nuclei stained with DAPI (blue). (Key: Scale bar = 20 nm; data representative of *n* = 3 independent experiments)

RHOQ was predominantly observed in vesicle-like structures within the cell, for instance in the filopodia, punctuate regions following trafficking routes within the cytoplasm and the peri-nuclear regions in control HUVECs (Fig. 7a). RHOQ expression increased and became predominately peri-nuclear in rhDLL4-stimulated cells (Fig. 7b).

*Exo70* mRNA (Fig. 5a) and Exo70 protein (Fig. 5bii) remained relatively constant in both rhDLL4-stimulated HUVECs and in RHOQ negative cells. Exo70 protein was present more evenly throughout the cell, as well as at the membrane in control cells (Fig. 7a). Upon rhDLL4 stimulation Exo70 protein became more clustered together and was also observed in the peri-nuclear regions of the cell (Fig. 7a). Some Exo70 co-localised with RHOQ positive vesicles in control EC and this co-localisation increased in rhDLL4-stimulated ECs (Fig. 7a).

**Fig. 8:**
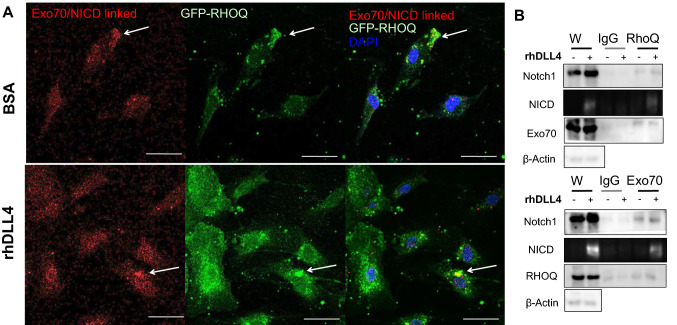
RHOQ/Exo70 co-localise with Notch components. **a** HUVECs were transfected with lentivirus to overexpress GFP tagged RHOQ and cultured on BSA or rhDLL4-coated plates and fixed 24 h later and Duo-link staining detecting only Exo70/NICD (red) associated antibodies, visualised by confocal microscopy. Nuclei stained with DAPI (blue). **b** Immunoprecipitation with either IgG control antibody, RHOQ antibody and Exo70 antibody and expression of Notch components assessed by western blotting, compared to whole cell (W) lysates, with B-Actin used as a loading control. (Key: White arrow, example of co-localisation; Scale bar = 20 nm; data representative of *n* = 3 independent experiments)

We then compared the localisation of Notch1 and the NICD to that of RHOQ and Exo70. Membranous and cytoplasmic staining of Notch1 was observed in control EC and in rhDLL4-stimulated cells Notch1 became more localised in peri-nuclear locations (data not shown). Interestingly, RHOQ and Exo70 co-localised with Notch1 in control EC (data not shown); increased co-localisation was observed for Notch1 with RHOQ and also Exo70 in rhDLL4-stimulated cells, particularly within the peri-nuclear area. NICD is barely detectable in BSA EC but found within the cytoplasm and nuclear expression in rhDLL4-stimulated EC samples (Fig. 7b); NICD also co-localised with RHOQ and Exo70 within the cytoplasm of the cells. Co-localisation of Exo70 and NICD proteins was further confirmed using dual labelling to highlight only proteins in close proximity to each other (Fig. 8a). GFP-labelled RHOQ strongly co-localised with the dual labelled Exo70/NICD proteins (Fig. 8a). Immunoprecipitation was used to confirm that RHOQ/Ex070 interacted with Notch1 and NICD (Fig. 8b). Exo70 was detected in IP pull downs with the RHOQ antibody and RHOQ was detected in reciprocal IP pull downs with the Exo70 antibody (Fig. 8b). Interaction with Notch1 and NICD was confirmed in IP pull downs with either RHOQ or Exo70 (Fig. 8b).

### Loss of RHOQ leads to abnormal localisation of Notch1 and blocked nuclear localisation of NICD

As RHOQ and Exo70 co-localised with Notch1/NICD we then investigated if RHOQ was involved in the process of trafficking Notch components and if the protein localisation was altered in RHOQ negative cells.

Exo70 localisation altered in RHOQ negative cells, forming larger clusters of proteins in both basal and rhDLL4-stimulated RHOQ negative cells, compared to controls (Fig. 7a). Interestingly, Notch1 also formed clusters of proteins in RHOQ negative cells, with more clusters forming near the nucleus in rhDLL4-stimulated negative cells (data not shown). The co-localisation of Exo70 and Notch1 in large clusters is more strongly observed in the RHOQ negative cells compared to controls in both basal and rhDLL4 (data not shown).

In the RHOQ negative cells the NICD was excluded from the nucleus, instead it was more clustered in the cytoplasm, particularly in rhDLL4-stimulated cells (Fig. 7b). The NICD still co-localised with Exo70, with stronger cytoplasmic association observed in the rhDLL4-stimulated RHOQ negative cells (Fig. 7b). To confirm this observation the NICD staining pattern was compared to BSA samples and rhDLL4-stimulated cells over a time course (Supplementary Fig. 4). Some cytoplasmic and clustered staining of NICD was observed, but predominantly the NICD staining became nuclear localised, particularly by 8 h rhDLL4-stimulated EC (Supplementary Fig. 4a). In siRHOQ negative cells, accumulation of the NICD was observed in BSA and rhDLL4-stimulated cells predominately in the cytoplasm, with stronger cytoplasmic staining observed in rhDLL4-stimulated cells by 16 h (Supplementary Fig. 4b).

**Fig. 9:**
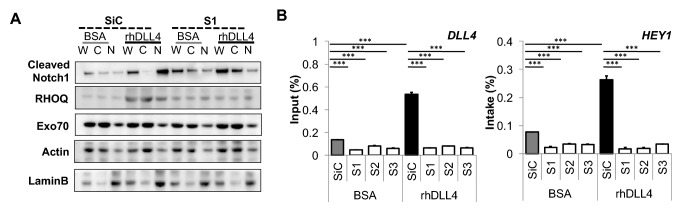
NICD does not translocate to the nucleus in RHOQ negative cells. **a** Transfected HUVECs with RHOQ siRNA duplexes were cultured on BSA or rhDLL4 (1 μg/ml)-coated plates for 8 h before (**a**) cells were pelleted and lysed for whole cell lysate (W), or cytoplasmic fraction (C), nuclear fraction (N) and changes in the NICD localisation assessed by western blotting, using B-Actin and Lamin B as markers of fraction contamination and loading control or being fixed and **b** analysed for NICD binding to the Notch promoter binding site for DLL4 and Hey1 by chromatin immunoprecipitation (Data representative of *n* = 3 independent experiments)

Cell fractionation was performed in parallel experiments to confirm localisation differences of the NICD (Fig. 9a). In control rhDLL4-stimulated cells, NICD accumulated in the nuclear fraction (Fig. 9a). However, in siRHOQ negative cells, although an increased NICD was observed in rhDLL4-stimulated cells, the NICD was predominantly present within the cytoplasm and not the nucleus (Fig. 9a). A ChIP assay further confirmed that the NICD was not translocated to the nucleus to stimulate downstream targets of DLL4/Notch signalling, as binding of the NICD to the promoters of *DLL4* and *HEY1* was not observed in siRHOQ negative cells (Fig. 9b).

### Loss of RHOQ expression leads to Notch1 degradation

**Fig. 10:**
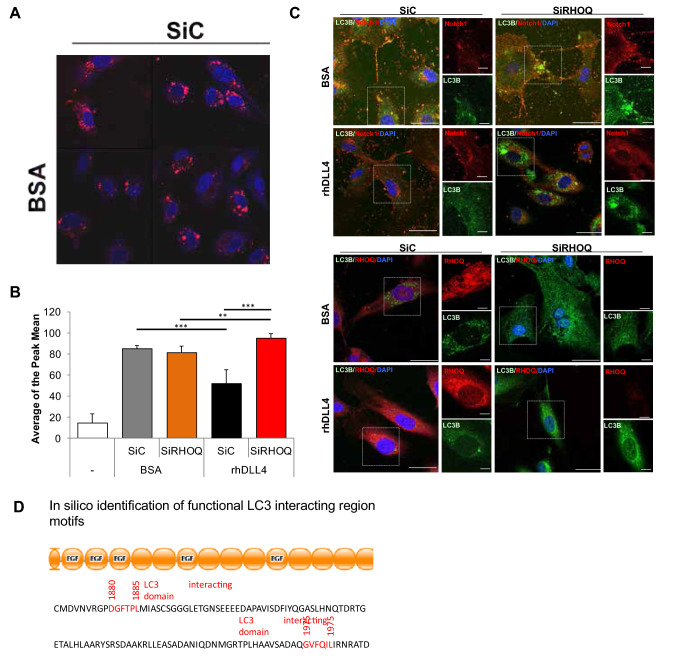
Loss of RHOQ increases autophagy and leads to degradation of Notch1 receptor. HUVECs cultured on BSA or rhDLL4 (1 μg/ml)-coated plates and effects on autophagosomes were assessed 16 h later by staining cells with autophagy tracker (red) with changes in tracker levels, **a** visualised by confocal microscopy (nuclei stained with DAPI (blue)), and assessed by **b** FACs or **c** cells were fixed and immuno-fluorescence staining for localisation changes in RHOQ (red) or Notch1 (red) and LC3B (green) proteins and visualised by confocal microscopy (nuclei stained with DAPI (blue)) or **d** illustrating LC3B binding sites on Notch1 (Error bars = S.D. Key: *p < 0.05, **p < 0.01, ***p < 0.0001 one-way ANOVA or unpaired Student’s *t*-test between data group and control, Scale bar = 20 nm; representative images and data of *n* = 3 independent experiments)

A further explanation for the decrease of the DLL4 and Notch1 is degradation of the internalised Notch1 receptor via autophagy [7]. In rhDLL4-stimulated cells compared to controls, autophagosome compartments decreased in number, as observed by identifying compartments using autophagy tracker and confocal microscopy (Fig. 10a) and FACs analysis (Fig. 10b). The opposite effect was observed in RHOQ negative cells, where the number of autophagosomes increased (Fig. 10a, b).

LC3B protein, recruited to the autophagosomes and leads to degradation of Notch1, also increased in both basal and hDLL4-stimulated RHOQ negative cells compared to controls, as observed by confocal microscopy (Fig. 10c). The co-localisation of Notch1 was compared to that of LC3B (Fig. 10c). In control basal and rhDLL4-stimulated cells some of the Notch1 present within the cytoplasm co-localised with LC3B (Fig. 10c). However, in rhDLL4-stimulated RHOQ negative cells, the majority of Notch1 present co-localised with LC3B (Fig. 10c). RHOQ did not co-localise with LC3B in control cells (Fig. 10c). The Notch1 receptor contains two possible LC3 binding domains in its C terminus as predicted, using the LIR database (https://ilir.warwick.ac.uk, Fig. 10d).

### The NICD is degraded in lysosomes of RHOQ negative cells

**Fig. 11:**
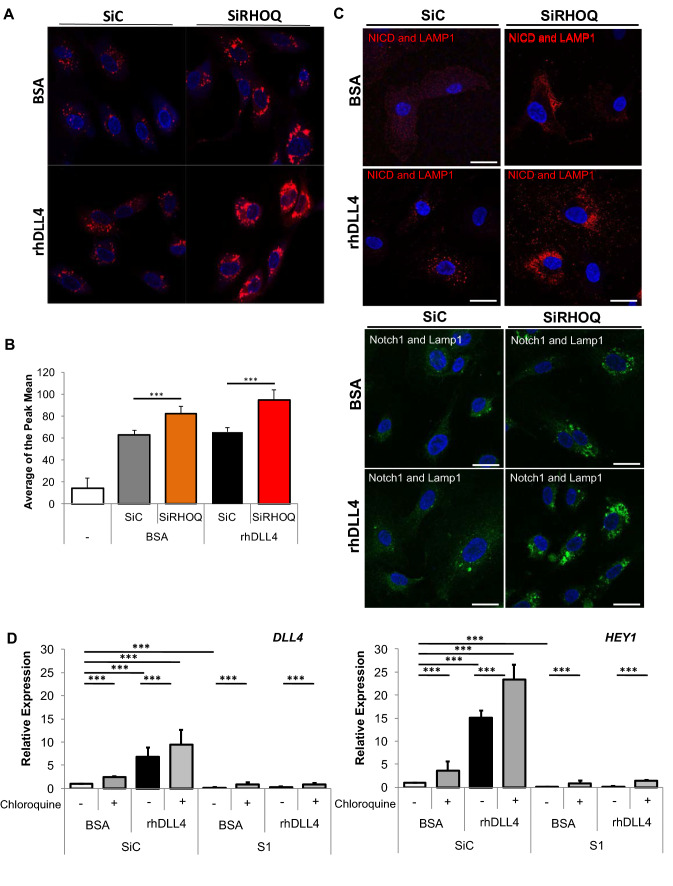
Loss of RHOQ expression leads to NICD degradation in lysosomes. HUVECs cultured on BSA or rhDLL4 (1 μg/ml)-coated plates and effects on lysosomes were assessed 16 h later by staining cells with lysosome tracker (red) with changes in tracker levels, **a** visualised by confocal microscopy (nuclei stained with DAPI (blue)) and assessed by **b** FACs or cells were fixed and immuno-fluorescence staining for localisation changes in proteins **c** duo-link staining detecting only Lamp/Notch1 (red) or Lamp1/NICD (green) associated antibodies, visualised by confocal microscopy. Nuclei stained with DAPI (blue). **d** Transfected HUVECs with RHOQ siRNA duplexes were cultured on BSA or rhDLL4 (1 μg/ml)-coated plates for 8 h with or without chloroquine (10 µM) before (**a**) changes in DLL4/Notch downstream target expression by QPCR (Error bars = S.D. Key: *p < 0.05, **p < 0.01, ***p < 0.0001 one-way ANOVA or unpaired Student’s *t*-test between data group and control, Scale bar = 20 nm; representative images and data of *n* = 3 independent experiments)

RHOQ protects CAL protein from being degraded in lysosomes by ensuring trafficking of CAL to the plasma membrane [25]. We investigated if RHOQ had a similar protective effect on degradation of the NICD and Notch1 (Fig. 11) using 16 h due strong cytoplasmic NICD localisation changes observed at this time point in RHOQ negative cells, by immuno-fluorescence (Supplementary Fig. 4). Using lysotracker to quantify the lysosomal compartments within the cells lacking RHOQ, we observed an increase in lysosomes in RHOQ negative cells, with a significant increase comparing rhDLL4 RHOQ negative cells to that of basal RHOQ negative cells by immuno-fluorescence (Fig. 11a) and by FACs analysis (Fig. 11b).

Lysosomal-associated membrane protein 1 (LAMP1) resides primarily across lysosomal membranes [38] and was used as another maker of lysosomes by immuno-fluorescence. LAMP1 increased in RHOQ negative cells, particularly within rhDLL4-stimulated negative cells (Fig. 11c). Notch1 co-localised with LAMP1 in some instances, which particularly increased in rhDLL4-stimulated RHOQ negative cells (data not shown); this interaction was further confirmed by dual labelling of Notch1 with LAMP1 (Fig. 11c). Co-localisation of NICD with LAMP1 was also observed particularly in rhDLL4 control-stimulated cells (data not shown). In siRHOQ negative rhDLL4 cells, there was a significant increase in the clusters of NICD in the cytoplasm, which co-localised with LAMP1 (data not shown). The increased close proximity of NICD and LAMP1 in RHOQ negative cells was further confirm by dual labelling techniques (Fig. 11c).

### Blocking autophagy and lysosomal pathways with chloroquine increased DLL4/Notch signalling in RHOQ negative cells

Chloroquine is widely used to inhibit lysosomal function as it becomes trapped in acidic compartments disrupts degradation by alkalinising the compartments [39]. Chloroquine significantly increased expression of downstream targets such as *DLL4* and *HEY1* in both basal and rhDLL4-stimulated control cells (Fig. 11d). Interestingly, 8 h exposure to chloroquine did increase the expression of *DLL4* and *HEY1* in both basal and rhDLL4-stimulated RHOQ negative cells (Fig. 11d).

## Discussion

Activation of DLL4/Notch signalling has been well documented to play a key role in the regulation of angiogenesis. However, the mechanisms downstream of ligand binding, such as transport to the nucleus and diversion down non-canonical pathways to degradation are less clear. We have identified RHOQ as a downstream target of DLL4/Notch signalling in endothelial cells, which plays a critical role in maintenance of Notch signalling, being essential for nuclear localisation and protection from degradation by non-canonical signalling.

The time frame of induction of the DLL4 gene downstream of DLL4/Notch signalling shows an oscillation with approximately a 6-h cycle [40] and we can see some indication of this in the western blots and staining of DLL4 and also RHOQ. There was a surprisingly uniform, closely timed induction of RHOQ. We propose that the early induction of RHOQ provides a feed-forward mechanism for enhancing and maintaining Notch signalling. RHOQ is already expressed in its active state basally, indicating its availability for amplification of DLL4 signalling.

The biological effects on vessel formation following loss or overexpression of RHOQ is consistent with the phenotype observed when the DLL4/Notch signalling pathway is compromised. Downregulation of basal DLL4 expression caused hyper-sprouting and inhibited the endothelial cell network formation [28], as did using soluble DLL4 to block Notch signalling [41] or inhibition of Notch signalling with a dominant-negative CSL construct [42]. Future studies should investigate whether the VEGF pathway is affected, as it is negatively regulated by Notch signalling [4].

Although RHOQ is a member of a multigene family, another Rho GTPase expressed by EC and involved in angiogenesis, RHOJ [43], could not compensate for the effect on the EC following loss of RHOQ within cells. RHOQ was found in activated form in basal conditions but increased by DLL4 activation of Notch. Therefore, some of the phenotype when suppressed is likely to be due to downregulation of DLL4 expression and hence blocking the feedback induction by DLL4.

Golgi-associated PDZ domain protein interacts with RHOQ to stabilise expression of β1-adrenergic receptor in intracellular compartments after internalisation, thus preventing the receptor from lysosomal degradation [44]. Notch1 is processed in the Golgi body and following cleavage is translocated to the plasma membrane [45]. Numb, as an example, regulates post-endocytic trafficking and degradation of Notch1 in lysosomes [46]. This shows a conserved role of RHOQ in intracellular trafficking of different proteins. Interestingly, components of the autophagy pathway, including autophagosomes and lysosomes, increased in rhDLL4-stimulated RHOQ negative cells. This represents another level of feed-forward control of Notch signalling by RHOQ. LC3B protein can be indicative of either increased autophagic flux (increased autophagosome formation and processing) or blocked autophagy due to lack of processing of the autophagosomes [47] and it would be interesting for future studies to continue assessing how loss of RHOQ influences the autophagy pathway.

Disrupting autophagy and lysosomal acidification can block NICD degradation, lead to increased NICD cleavage and also intensify Notch signalling [48–51]. The ability of chloroquine to rescue RHOQ suppressed cells supports the potential role of RHOQ in targeting to this compartment.

**Fig. 12:**
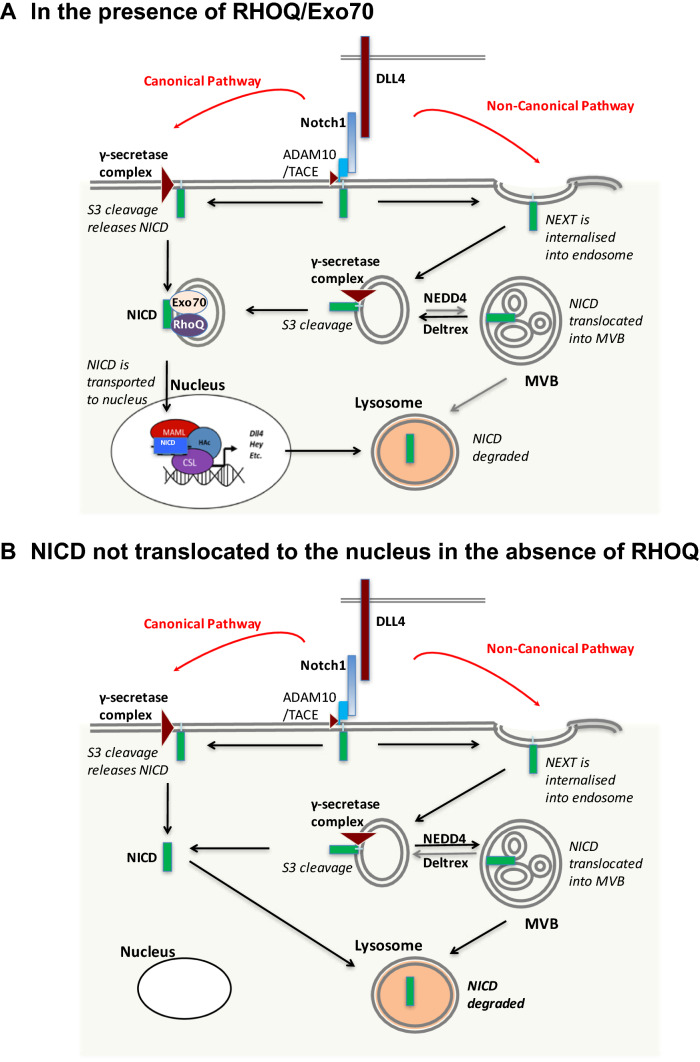
Summary: RHOQ regulates Notch signalling by transporting cleaved Notch1 to the nucleus, otherwise Notch1 is degraded. **a** Binding of Notch1 with DLL4 leads to cleavage of Notch1 by ADAM/TACE complex (generating the Notch extracellular truncation (NEXT). Membrane bound gamma-secretase leads to the release of the NICD. The NICD combines with the RHOQ/Exo70 positive nucleus bound endosome, which transports the NICD to the nucleus and subsequent modulation of downstream targets of DLL4/Notch signalling. **b** In RHOQ negative cells NICD accumulate within the cytoplasm. Loss of translocation of the NICD to the nucleus prevents the induction of the downstream targets of Notch signalling within the nucleus. Rather the NICD is targeted for degradation in lysosomes

In summary, we propose that after DLL4 binding to Notch1 receptor (Fig. 12), cleavage of the Notch1 receptor and release of the NICD leads to interaction with Exo70. Basally active RHOQ interacts with the Exo70/NICD. These RHOQ positive vesicles in conjunction with Exo70/NICD translocate the NICD to the nucleus. *RHOQ* expression induced by NICD ensures that sufficient RHOQ is present within the cell after DLL4/Notch signalling. Reduced RHOQ expression leads to the sequestering of the NICD within the cytoplasm and degradation in lysosomes, and thereby prevents transcription of downstream targets. This included *DLL4*, leading to reduced ligand DLL4 expression levels on the cell surface, and thereby further reducing the magnitude of the DLL4/Notch signalling within endothelial cells. Loss of DLL4/Notch signalling in RHOQ negative cells is further compounded by the accumulation of Notch1 receptor within the cytoplasm of cells, leading to degradation via the autophagy pathway.

Thus, we hypothesise RHOQ mediates a critical balance essential for Notch signalling involved in an amplification loop in endothelial cells in vivo and in vitro and also regulates the division between canonical and non-canonical signalling. This may also provide a mechanism for the oscillatory response to Notch signalling and rapid changes in tip and stalk signalling [40].

## Methods

### Cell culture

HUVECs (to passage 6; Lonza, Wokingham, UK) were cultured in EGM-2 (Lonza). Mouse EC lines SEND and SVEC140 were cultured in 10% FCS containing DMEM (Invitrogen).

### Inhibition of Notch signalling

HUVECs were seeded onto dishes pre-coated with BSA (1 μg/ml; Sigma-Aldrich) or rhDLL4 extracellular domain (1 μg/ml; R&D Systems, Minneapolis, USA) in 0.2% (w/v) gelatin (Sigma-Aldrich). The γ-secretase Notch inhibitor Dibenzazepine (DBZ; Sigma-Aldrich; 20 nM; vehicle control DMSO; Sigma-Aldrich) was added at time of seeding.

### siRNA transfection

HUVECs were transfected with negative medium stealth duplex (siControl; Invitrogen), or RHOQ siRNA duplexes, referred to also as siRHOQ (see Supplementary Table 1 for sequences) using RNAiMaxi Lipofectamine (Invitrogen) at a final concentration of 0.3% (v/v). Transient loss of RHOQ mRNA remained reduced by at least 60% over a three-day period compared to controls (data not shown).

### Stable modulation of RHOQ expression

For overexpression, RHOQ and GFP-RHOQ were cloned into pLenti 6.2/V5-DEST (Invitrogen). For stable knockdown, MISSION shRHOQ plasmids were purchased (Invitrogen; clone numbers TRCN0000289568 and TRCN0000047588). All plasmid inserts were packaged into virus using HEK293 cells and the ViraPower™ packaging mix (Invitrogen), according to manufacturer’s instructions.

### RNA extraction, reverse transcription and real time quantitative PCR (QPCR)

RNA was extracted using TriReagent (Sigma-Aldrich) and complementary DNA was synthesised using a High Capacity cDNA Reverse Transcription Kit (Life Technologies) following manufacturers protocol. Real-time quantitative PCR (qPCR) was carried out using the sensiMix Syber No-Rox One-Step kit (Bioline) on a 7900HT Fast qPCR System. Relative quantification was done using the ΔΔCt method normalising to relevant house-keeping genes (HKG); see Supplementary Table 1 for sequences.

### Chromatin immunoprecipitation (ChIP) assay

ChIP analysis was performed using the EZ-ChIP™ kit (Millipore) following manufacturer’s protocol. Lystates were incubated with antibodies [Notch1 (3608, Rabbit), Immunoglobulin G (Rabbit IgG); Cell signalling] and purified samples were analysed by QPCR (see Supplementary Table 1 for primers). Raw Ct values were analysed using the formula 100 × 2^((Ct input − log2(100)) − Ct IP)^ to obtain percent input values. Cells were incubated in the dark for 15 min at RT before 400 μl of PBS was added and analysis of cell populations by FACS-Becton Dickson FACsCaliber (Becton and Dickson, USA). 10,000 cells were analysed per condition. Cell Quest Software (v.3.1f) displayed the results as a bivariate dot plot of Annexin V and PI fluorescence intensity*.*

### Western blots

Cells were lysed in RIPA buffer (Sigma-Aldrich) containing Complete Mini Protease Inhibitor (Roche) and 1 × Phosphatase Inhibitor 2&3 (Sigma-Aldrich). Lystates were mixed with 2 × SDS loading buffer and boiled at 95 °C for 10 min. Protein samples were separated on a 4–12% gradient SDS Page Gel (Invitrogen) and transferred to a polyvinylidene difluoride membrane before blocked in 5% Bovine Serum Albumin. Membrane were probed with primary antibodies used were RHOQ (T8950, Rabbit; Sigma-Aldrich), and Notch1 (3608, Rabbit), Cleaved Notch1 NICD (2421, Rabbit), DLL4 (2421, Rabbit), VEGFR2 (2479, Rabbit), phosphVEGFR2 (2478, Rabbit), B-Actin (3700, Mouse) from Cell signalling and Exocyst70 (sc-365825, Mouse), Cleaved Notch1 (sc-23307, Goat) from Santa-Cruz. Horseradish peroxidase (HRP)-conjugated secondary Antibodies (DAKO) were used and labelled proteins detected using chemiluminescence (ECL) reagent or ECL prime (GE Healthcare) and imaged using ImageQuant LAS 4000 (GE Healthcare). Describe in detail domains recognised by Notch 1 ab and cleaved Notch 1 ab-how did this distinguish the 2 forms-critical for paper-needs to be very clear.

### Immunoprecipitation

Immunoprecipitation was carried out according to manufactures protocol (Pierce Crosslink Immunoprecipitation Kit, Fisher Scientific). Briefly, RHOQ (Sigma) or Exocyst70 (Abcam) antibody was permanently coupled to Protein A/G plus Agarose Resin and incubated with protein lystates. The antigen was then eluted and detected following western blotting principle. TrueBlot HRP-Secondary anti-rabbit or anti-mouse Antibodies (Rockland, USA) were used to minimise detection of antibody large or small chain fragments.

### GTPase activation assay

A GTPase activation assay kit (Cambridge Bioscience Ltd) was used to selectively precipitate the active form of RHOQ from HUVEC treated ± rhDLL4 over a time course of 24 h. Equal amounts of protein were used in the assay. RHOQ was then detected by western blot techniques.

### 3D in vitro hanging drop sprouting angiogenesis assay

EC spheroids were generated by the hanging drop method [52]. Briefly, control, siRNA-treated or stably infected spheroids were solidified in 200 μl of Reduced Matrigel BD (Reduced growth factor containing matrigel; BD Biosciences, Nottingham) or into Matrigel containing siRNA duplex targeting RHOQ or siControl duplex (0.5 μM) and cells cultured as normal.

### Matrigel tube formation assay

siRNA or stably infected HUVECs were cultured on top of 100 μl of Reduced Matrigel BD (BD Biosciences). Changes in tube formation were quantified by counting the number of branch points (by 3 or more cells) or number of quadrants (enclosed areas) from 10 fields of view from each condition.

### Chicken chorioallantoic membrane (CAM) assay

White fertilised eggs (Henry Stewart & Co Ltd (Lincolnshire), delivered and stored at 4 °C, classed as egg development day (EDD) 1. Briefly, eggs were incubated at 37.5 °C. From EDD1-3 eggs were placed on a 90° tilting rack (rotating > 6 × /24 h). From EDD3 eggs were placed upright and a small hole made in egg shell. A 1 cm diameter plastic ring was placed on the surface of the CAM on EDD6 of fertilised eggs and eggs infected with 100 μl of control, shRHOQ, or overexpressing hRHOQ viruses. On EDD10 or just prior to EDD13.9 the eggs were placed at 4 °C to induce hypothermia, 1 ml of 10% zinc oxide/vegetable oil (Sigma-Aldrich) injected underneath the plastic ring to visualise vasculature. All work was completed prior to the point at which embryos become a protected animal under the Home Office guidelines. Images were quantified using HetCAM software to determine the normalised vessel area and length of vessels.

### Retinal angiogenic model

Eyes were obtained from C57BL/6 pups aged between day 3 to 15 old (Charles River). 0.5 μM in 1 μl siRNA targeting RHOQ [5′-CAGUACCUCUUGGGACUCUAUGACA-3′] GFP-labelled JetPEI in vivo delivery system (VWR), prepared according to manufactures protocol, was injected into the eyes of day 3 old pups and the eyes were harvested three days later. Harvested eyes were fixed overnight in 4% formation/PBS at 4 °C before being dissected to obtain the retina, according to methodology outlined previously [53]. Briefly, retinas were permeabilised in ice-cold methanol for 1 h at − 20 °C, washed with PBS 0.1% Tween, blocked with 2.5% horse serum and visualised following the immuno-fluorescence protocol outlined previously. ImageJ was used to quantify vascular network changes on representative low × 10 images. All work was conducted in accordance with the UK Home Office guidelines, under the project licence PPL30-3197.

### Immuno-fluorescence

siRNA transfected cells were either grown as monolayer on pre-coated with BSA or rhDLL4 extracellular domain on cover slips. Up to 24 h later, cells were rinsed in PBS, fixed in 4% buffered formalin and permeabilised by incubating samples at − 20 °C in ice-cold methanol for 20 min. Samples were washed in PBS before blocking in 2.5% horse serum in PBS (Sigma-Aldrich) for 1 h and incubated with primary antibodies diluted in 2.5% horse serum in PBS O/N; Antibodies used were RHOQ (T8950, Rabbit; Sigma-Aldrich), Notch1 (3608, Rabbit), Cleaved Notch1 NICD (2421, Rabbit), from Cell Signalling and Exocyst70 (sc-365825, Mouse), Cleaved Notch1 (sc-23307, Goat) from Santa-Cruz. Secondary detection was achieved using donkey anti-rabbit/goat/mouse were IgG labelled with Alexa Flour 488/594/647 (Life technologies). Samples were incubated in 1 μM Hoechst (Sigma-Aldrich) for 5 min to visualise the nucleus. Confocal imagines were captured on a Zeiss 780 Inverted Confocal microscope. Scale bars were added to images using ImageJ.

### Dual labelling immuno-fluorescence

Duo-link is based on in situ proximity ligation assay, where the secondary fluorescent tagged antibody can only bind to linked primary antibodies bound to two different target epitopes (a theoretical maximum distance being 10 nm to be able to create a signal). Duo-link staining (Sigma) was carried out according to manufactures protocol. Briefly, siRNA transfected cells on cover slips were blocked in blocking solution, incubated with primary antibodies (see above), washed in buffer provided before being incubated in corresponding PLA probes for 1 h at 37 °C. Samples were then washed and primary antibodies in close proximity were then linked together during the ligation step, by incubating cells with ligation solution for 30 min at 37 °C. After washing the signal was amplified by incubating cells with the polymerase for 100 min at 37 °C. Samples were washed in buffers provided and incubated in 1 μM Hoechst (Sigma-Aldrich) for 5 min to visualise the nucleus. Confocal imagines were captured on a Zeiss 780 Inverted Confocal microscope. Scale bars were added to images using ImageJ.

### Autophagy and lysosome tracker

Analysis of changes in lysosome (LysoTracker Red, ThermoFisher Scientific) and autophagy (Autophagy Sensor LC3B-RFP, ThermoFisher Scientific) compartments was carried out according to manufactures protocol. Briefly, siRNA transfected cells were either grown as monolayer on pre-coated with BSA or rhDLL4 extracellular domain, as previously described in culture dishes (for flow cytometry) or cover slips (Immuno-fluorescence) for 16 h. Media was then removed and cells incubated in lysotracker (50 nM) or autophagy tracker (~ 1** × **10^8^ particles/ml) in the incubator in the dark for 30 min.

#### Flow cytometry

Adherent cells were trypsinised, suspended in 2.5% horse serum in PBS and analysis of 10,000 cells by FACS-Becton Dickson FACsCaliber (Becton and Dickson, USA). Lysotracker was detected using absorbance 577 nm and emission on 590 nm or autophagy was detected for Red Fluorescence protein (550 nm and 600 nm). Cell Quest Software (v.3.1f) displayed the results fluorescence intensity to calculate peak mean*.*

#### Immuno-fluorescence

In the dark, cells were rinsed in PBS, fixed in 4% buffered formalin, washed in PBS before incubated in 1 μM Hoechst (Sigma-Aldrich) for 5 min to visualise the nucleus and mounted onto glass slides. Confocal imagines were captured on a Zeiss 780 Inverted Confocal microscope. Scale bars were added to images using ImageJ.

### Statistical analysis

All statistical analysis was carried out using GraphPad Prism (v6.0) by unpaired Student’s *t*-test or one-way ANOVA followed by Tukey’s multiple comparisons test on independent experimental replicates, unless otherwise indicated. A *P*-value < 0.05 was considered statistically significant.

## Electronic supplementary material

Below is the link to the electronic supplementary material.

Supplementary file1 (DOCX 17 kb)

Supplementary file2 (PDF 974 kb)

Supplementary file3 (DOCX 135 kb)
