# Supplementary material for: RHOQ is induced by DLL4 and regulates angiogenesis by determining the intracellular route of the Notch intracellular domain
Source: Angiogenesis. 2020 Jun 6;23(3):493–513. doi: 10.1007/s10456-020-09726-w (PMC7311507; doi:10.1007/s10456-020-09726-w)
Supplement: Supplementary file 1 — Supplementary file1 (DOCX 17 kb) [file 10456_2020_9726_MOESM1_ESM.docx]

**Sup Fig 1. Modifying RHOQ expression effected angiogenic processes *in vitro*.** A) HUVECs were transfected with siControl (SiC) or RHOQ siRNA duplexes (concentration 20nM; S1, S2, S3) for transient loss or infected with B) control (shC) virus or RHOQ shRNA duplexes (sh1, sh2) containing virus for stable loss of RHOQ. HUVECs were then grown on BSA or rhDLL4-coated plates and harvested after 24h to confirm changes in RHOQ expression by i) QPCR and expressed relative to the BSA control cells or ii) western blotting, using B-Actin as a loading control. Densitometry was performed on western blots and the ratios relative to the 4h BSA sample. Representative images and quantification of phenotypic changes in C) the hanging drop sprouting assay or D) in the tube formation assay using transfected siRHOQ HUVECs compared to control siC cells. [Error bars = S.D. Scale bar = 20nm. Key: * p<0.05, ** p<0.01, *** p<0.0001 one-way ANOVA or unpaired Student’s t-test comparing two data groups; data representative of n=3 independent experiments]

**Sup Fig 2. Modifying RHOQ expression disrupted developing vasculature in the chicken CAM assay.** A) Chicken fibroblasts were infected with control (hC) virus or human RHOQ (hRHOQ) virus to overexpress hRHOQ and were harvested after 24h to confirm changes in RHOQ expression by QPCR. Images and analysis of vasculature changes on developmental day B) 10 and C) 13.9 following exposure to hC or hRHOQ virus. Key: Scale bar 100nM; Error bars = S.D. Key: * p<0.5, ** p<0.1, *** p<0.001 unpaired Student’s t-test comparing two data groups; data representative of n=3 independent experiments.

**Sup Fig 3. Overexpression of RHOQ does not change surface expression of Notch components.** HUVECs stably expressing human RHOQ (hRHOQ) were assessed for the effects on DLL4 or Notch1 expression by A) staining cells for surface protein surface expression, analysed by FACs and expressed relative to control, with example representative image provided, B) membrane fraction by western blotting, using CD31 as a loading control or C) by QPCR, expressed relative to GAPDH. [Error bars = S.D. Key: * p<0.05, ** p<0.01, *** p<0.0001 one-way ANOVA or unpaired Student’s t-test between data group and control; data representative of n=3 independent experiments]

**Sup Fig 4.** NICD accumulates in cytoplasm in RHOQ negative cells. HUVECs were transfected with A) siControl duplex (SiC) or B) RHOQ siRNA duplex S1 (concentration 20nM) and cultured on BSA or rhDLL4 (1μg/ml)-coated plates and fixed over a time course before immuno-fluorescence staining for NICD (green), visualised by confocal microscopy. Nuclei stained with DAPI (blue). [Key: Scale bar = 20nm; data representative of n=3 independent experiments]

**Sup Table 1**. siRNA, QPCR and CHIP sequences
