# Supplementary figures and images for: RHOQ is induced by DLL4 and regulates angiogenesis by determining the intracellular route of the Notch intracellular domain
Source: Angiogenesis. 2020 Jun 6;23(3):493–513. doi: 10.1007/s10456-020-09726-w (PMC7311507; doi:10.1007/s10456-020-09726-w)

Sup Fig1

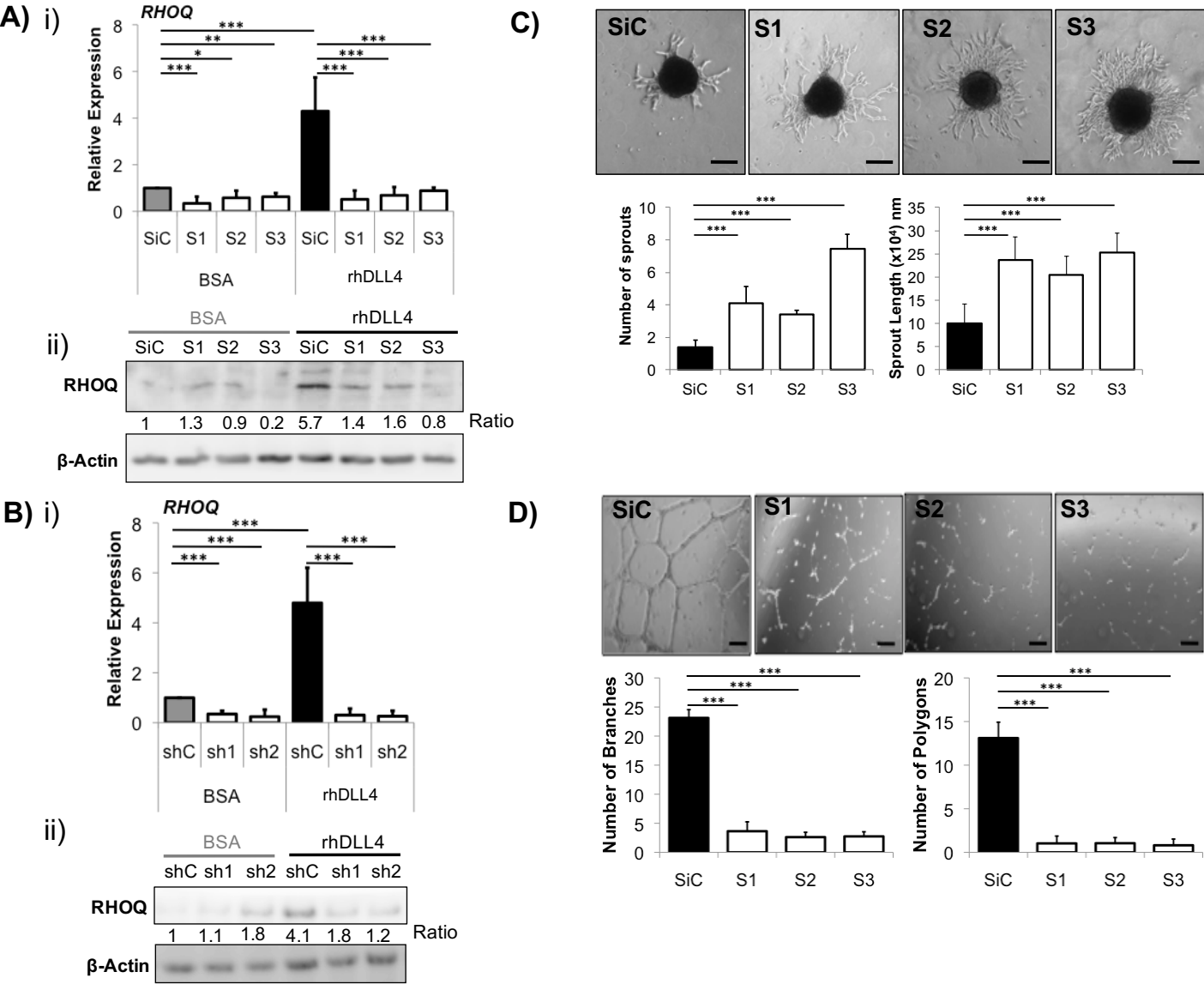

Sup.Fig2

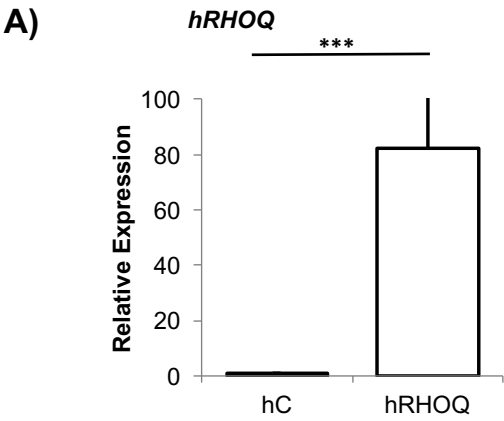

**B) Day 10**

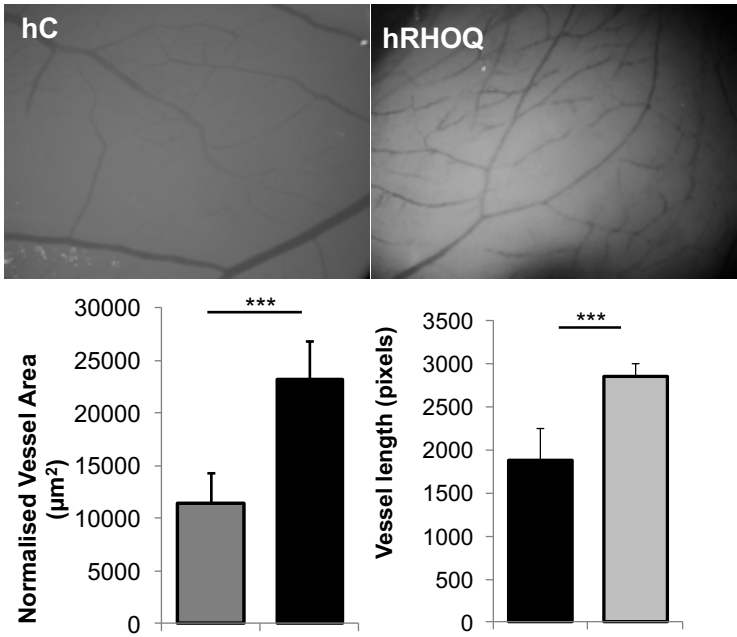

**C) Day 14**

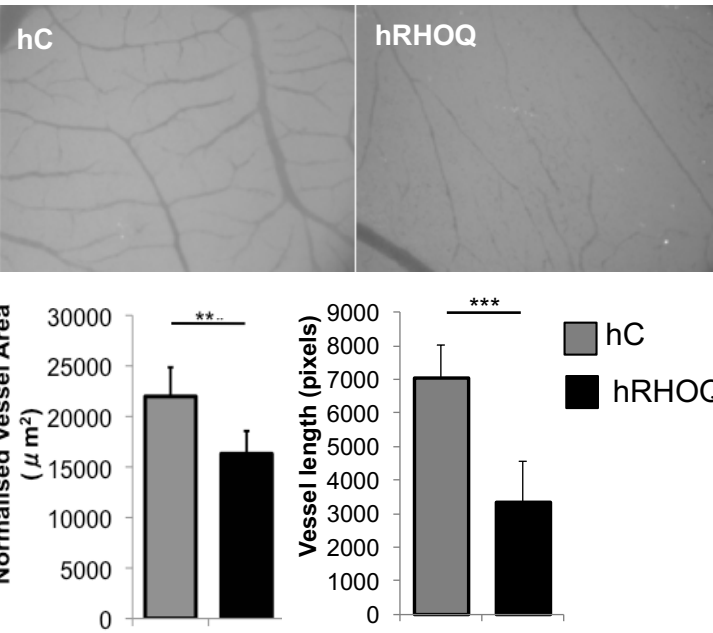

**Sup.Fig3**

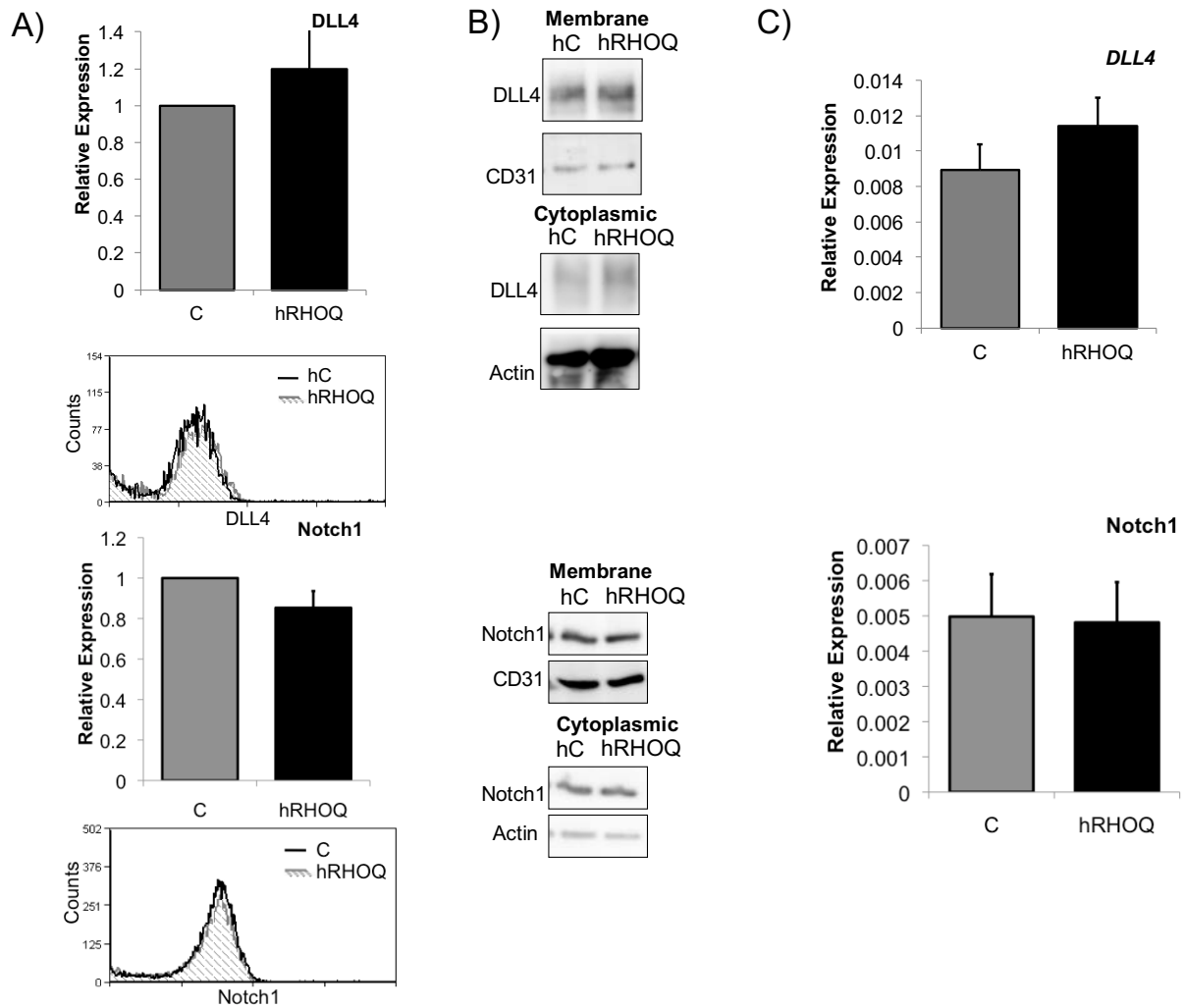

Sup.Fig4

A) SiC

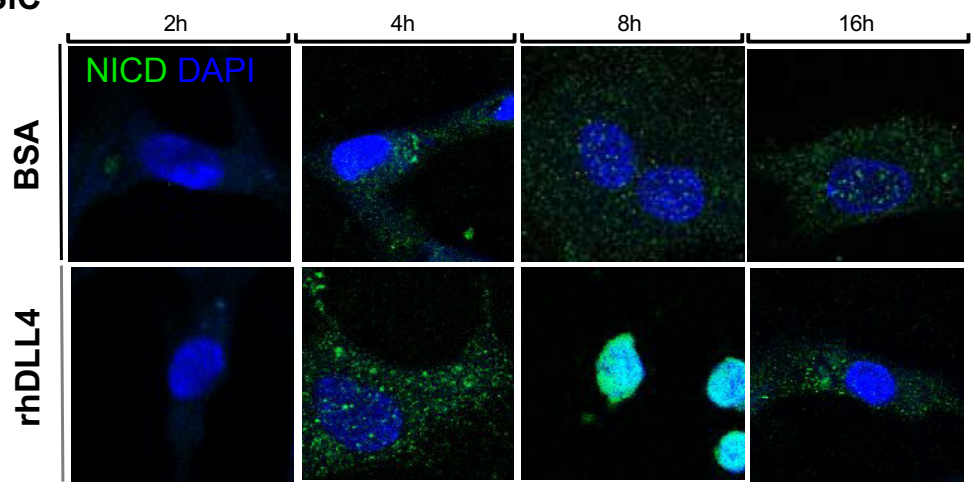

B) SiRHOQ

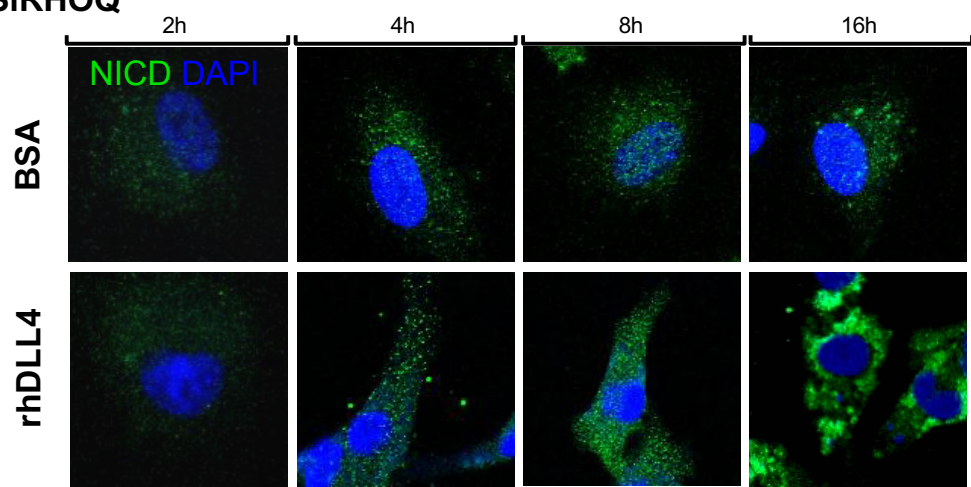

Supplement: Supplementary file 2 — Supplementary file2 (PDF 974 kb) [file 10456_2020_9726_MOESM2_ESM.pdf]
