# Supplementary material for: RHOQ is induced by DLL4 and regulates angiogenesis by determining the intracellular route of the Notch intracellular domain
Source: Angiogenesis. 2020 Jun 6;23(3):493–513. doi: 10.1007/s10456-020-09726-w (PMC7311507; doi:10.1007/s10456-020-09726-w)
Supplement: Supplementary file 3 — Supplementary file3 (DOCX 135 kb) [file 10456_2020_9726_MOESM3_ESM.docx]

| siRNA duplex sequences | hRHOQ siRNA duplex 1 | | 5’-TGACTGGGATTTGGTTTCCTCATTA-3’ |
| --- | --- | --- | --- |
|  | hRHOQ siRNA duplex 2 | | 5’-GGTAGAACAGAGATCTCCAACGTCT-3’ |
|  | hRHOQ siRNA duplex 3 | | 5’-AGGTTAGCTCATGGTGAATTCTATT-3’ |
|  | mRHOQ siRNA duplex 1 | | 5’-CAGUACCUCUUGGGACUCUAUGACA-3’ |
|  | mRHOQ siRNA duplex 2 | | 5’-GAGUGGGUACCAGAGCUAAAGGAAU-3’ |
|  | mRHOQ siRNA duplex 3 | | 5-GAACUCAGAUUGAUCUCCGAGAUGA-3’ |
| qPCR primers | *hGAPDH* | Forward | AGCCACATCGCTCAGACAC |
|  |  | Reverse | GCCCAATACGACCAAATCC |
|  | *hHEY1* | Forward | CGAGCTGGACGAGCCCAT |
|  |  | Reverse | GGAACCTAGAGCCGAACTCA |
|  | *hHES1* | Forward | AGTGAAGCACCTCCGGAAC |
|  |  | Reverse | CGTTCATGCACTCGCTGA |
|  | *hDll4* | Forward | CCCTGGCAATGTACTTGTGAT |
|  |  | Reverse | TGGTGGGTGCAGTAGTTGAG |
|  | *hRHOQ* | Forward | acaaaataccggcttccaga |
|  |  | Reverse | tcaacattaattacttctagcctgatg |
|  | *hNotch1* | Forward | CGCACAAGGTGTCTTCCAG |
|  |  | Reverse | AGGATCAGTGGCGTCGTG |
|  | *hVEGFR1* | Forward | GCACCTTGGTTGTGGCTGAC |
|  |  | Reverse | CGTGCTGCTGCTTCCTGGTCC |
|  | *hVEGFR2* | Forward | GAACATTTGGGAAATCTCTTGC |
|  |  | Reverse | CGGAAGAACAATGTAGTCTTTGC |
|  | *mGAPDH* | Forward | AAGGCCAACCGTGAAAAGA T |
|  |  | Reverse | GTGGTACGACCAGAGGCATAC |
|  | *mRHOQ* | Forward | GAATCTAAGCGCGTCCTGTG |
|  |  | Reverse | CCAAGCGGACATCAGTTTTAC |
| CHIP QPCR primers | *hDll4* | Forward | gacgcttagcttggcctggagctg |
|  |  | Reverse | tgtaaaatacaggaaggggc-ccgtcag |
|  | *hHEY1* | Forward | AATTCAGCGGCGCGAGA |
|  |  | Reverse | CTCACGCTTTGCCTCTGGTTA |
|  | *hRHOQ* | Forward | tgcctcagag-ttatcgtaaaaacc |
|  |  | Reverse | tggtagcagtgtggggatg |

## Supplementary Table 1
